# New Intraspinal cause of physiological FDG uptake

**DOI:** 10.4103/0972-3919.78257

**Published:** 2010

**Authors:** S Padma, Sundaram P Shanmuga, GS Shagos, Harish S Vijay

**Affiliations:** Department of Nuclear Medicine and PET CT, Amrita Institute of Medical Sciences and Research Center, Cochin, Kerala, India

**Keywords:** Artery of Adamkiewicz, Dedifferentiated thyroid cancer, FDG PET scan, papillary Ca thyroid, radioiodine I 131 scan, thyroglobulin

## Abstract

We present a paediatric case of Papillary Ca thyroid under evaluation for elevated Thyroglobulin (Tg) level with negative ^131^I wholebody scintigraphy. Differentiated thyroid cancer (DTC) arises from follicular epithelium and retains basic biological features like expression of sodium iodide symporter (NIS), which is the cellular basis of radio iodine (^131^I) concentration during thyroid ablation. Once dedifferentiation of thyroid cells occurs, cells fail to concentrate ^131^I, posing both diagnostic and therapeutic problems in DTC and one may have to resort to other imaging techniques for disease localization. As DTC progression is slow, patients have a relatively good prognosis. However children with thyroid malignancies need aggressive management, as initial presentation itself maybe with nodal metastases. It is well known that FDG PET CT apart from its oncological applications, is also used in the evaluation of vascular inflammation especially Takayasu’s arteritis. It is also reported in literature, that ^18^F-FDG uptake can be seen relatively frequently in the arterial tree of cancer patients. Dunphy et al reported the association of vascular FDG uptake in inflammation as well as in normal arteries. This study typically describes FDG uptake in a patchwork of normal vessel, focal inflammation and or calcification of vessels. The other plausible reasons for significant vascular ^18^F-FDG uptake are drugs such as potent non steroidal anti-inflammatory agents, dexamethasone, prednisone and tacrolimus. Our patient showed false positive ^18^F Fluorodeoxyglucose (FDG) uptake in spinal cord at D11/12 and D12/L1 vertebral levels in FDG PET CT imaging performed as part of raised Thyroglobulin workup. This intra spinal FDG uptake is attributed to physiological uptake and inadequate FDG clearance from artery of Adamkiewicz, which can be added as a new physiological cause of FDG uptake unreported in literature as yet.

## INTRODUCTION

^18^F Fluorodeoxyglucose (FDG) Positron emission tomography (PET) is increasingly performed in DTC patients presenting with elevated serum Tg levels and negative ^131^I scan.[1] With more and more centres acquiring PET CT scanners, it is imperative for the imageologist to know the potential pitfalls and artifacts of FDG imaging. Nowadays FDG PET CT imaging is routinely used in the evaluation of vascular inflammatory pathologies like Takayasu’s arteritis. In these cases, pathological vessel wall FDG uptake is due to macrophage activation and cellular proliferation.[2,3] Physiological FDG uptake occurs in many sites of the body and may cause confusion in interpretation particularly in oncological setup. Clinical correlation, awareness of physiological FDG uptake sites and FDG avid benign lesions are necessary to avoid potential pitfalls in image interpretation.

## CASE REPORT

**Figure 1:**
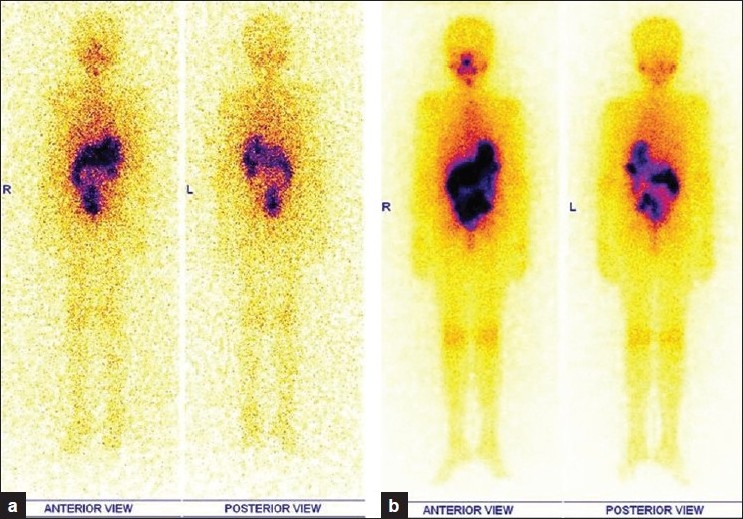
a) Followup Whole body ^131^I scan in 2008 (in anterior and posterior projections) 6 months post I 131 therapy showing persistent minimal I 131 uptake in anterior neck; b) Post therapy whole body ^131^I scan, 2^nd^ dose (in anterior and posterior projections) showing focal I 131 uptake in anterior neck as expected in an immediate post therapy setting with no new focal spots elsewhere

Our patient is an 8-year-old girl who presented to the Head and Neck department with swelling in lower neck noticed by her mother 10 days back. She had no history of pain, dysphagia, dyspnea, hoarseness of voice, cough, hemoptysis or bone pain. There is no history of hyper or hypothyroidism. Local examination revealed a 2 × 2 cm well defined, mobile nodule in midline region of thyroid, moving with deglutition. There were multiple small palpable bilateral cervical nodes. FNAC of thyroid was reported as Papillary carcinoma thyroid. Preoperative Serum Tg was 91.95 ng/ml. Total thyroidectomy with bilateral neck lymph nodal dissection (level II to V) was done in 2007. Histopathology confirmed the diagnosis of papillary carcinoma thyroid with bilateral cervical lymph nodal metastases. Patient underwent a residual thyroid and whole body ^131^I scan which showed moderate residual thyroid tissue. Patient was treated with 925 MBq (Megabecqueral) of ^131^I sodium iodide solution orally. A follow up whole body ^131^I scan in October 2008 showed persistence of minimal residual thyroid tissue [Figure 1a]. Serum Tg off Thyroxin was 18.28 ng/ml (normal values in intact thyroid pt 1.4 to 78.0), Tg antibody was 3.20 IU/ml (normal is less than 225).

Based on Whole body ^131^I scan findings and raised Tg, patient was taken up for second sitting of high dose ^131^I therapy [Figure 1b]. She was orally treated with 1406 MBq of ^131^I sodium iodide solution

**Figure 2:**
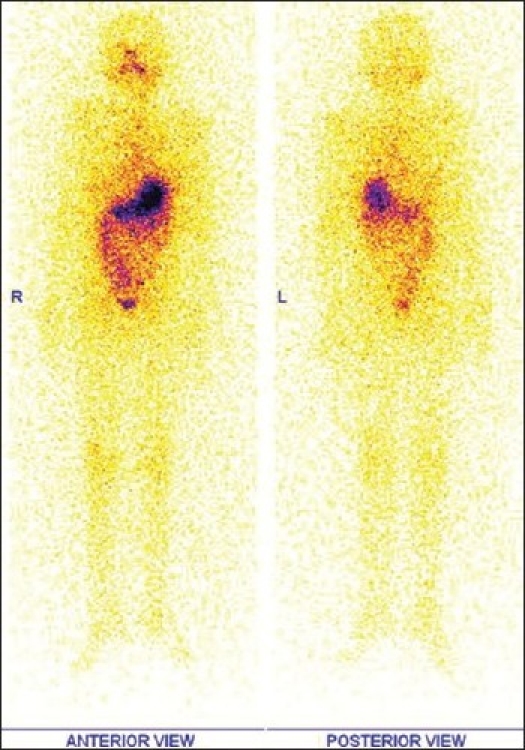
Followup Whole body ^131^I scan in 2009 (in anterior and posterior projections) showing no abnormal I 131 uptake in anterior neck as well as in rest of whole body survey

Follow-up Whole body ^131^I scan in 2009, showed no abnormal uptake in neck as well as in rest of whole body survey [Figure 2]. Tg off Thyroxine showed elevated values of 21 and 23 ng/ml respectively, done at intervals of two months. Ultrasound neck and chest X-Ray were non-contributory.

**Figure 3:**
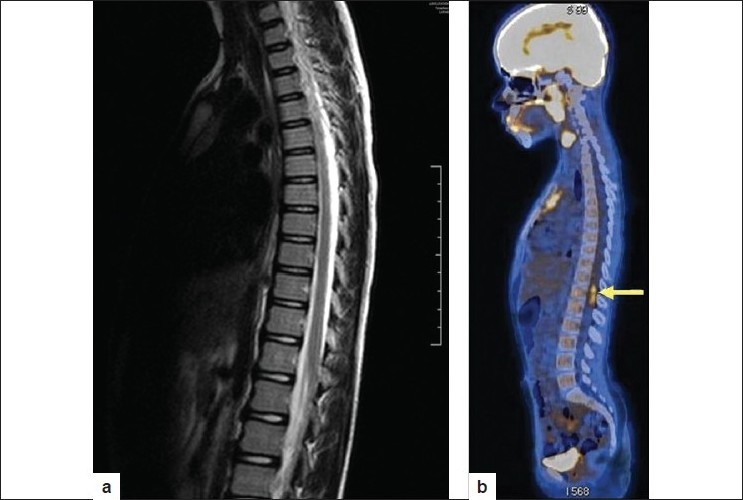
a) Sagittal view MR of Lumbosacral spine showing no abnormal signals; b) Sagittal view FDG PET CT showing linear focus of FDG uptake in D11/12 vertebral level

**Figure 4:**
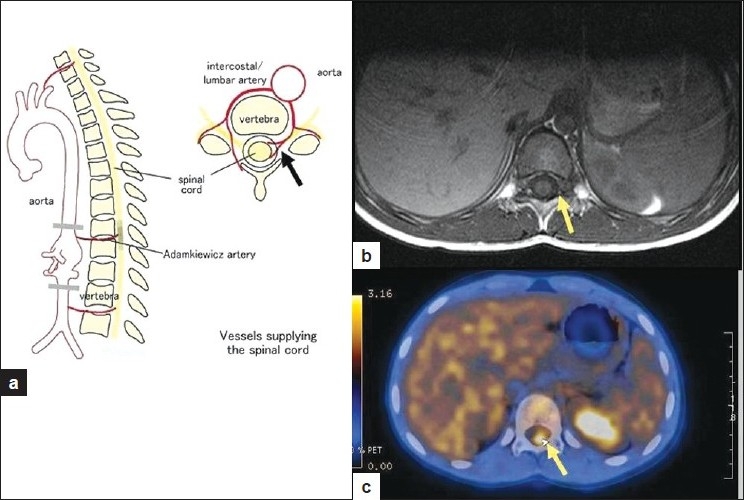
a) Showing pictorial representation of vessels supplying Spinal cord in sagittal and transaxial views. The Adamkiewicz artery is the largest segmental medullary artery typically arises from a left posterior intercostal artery, which branches from aorta and supplies two thirds of the spinal cord via anterior spinal artery. In 75% of people, the artery of Adamkiewicz originates on the left side of the aorta between the D9 and D11 vertebral segments. In approximately 30% of people it arises from the right side. 25% of population can have two large anterior segmental medullary arteries; b) MR Transaxial view showing no altered signals in D11/D12 levels; c) Transaxial view of FDG PET CT depicting abnormal FDG uptake intraspinally at D11/12 vertebral level (SUV Max is 3.95 g/ml). The branching of vessels supplying spinal cord have been highlighted with arrow in all three images

As part of the workup, a Whole body PET CT scan was performed (off Thyroxin) which showed an abnormal linear focus of FDG uptake in intraspinal region of D11/12 and D12/L1 vertebral levels with a Standard Uptake Value (SUV) of 3.95 g/ml. There was no abnormal FDG uptake elsewhere in neck and rest of whole body survey. Subsequent MR spine showed no abnormality at lower dorsal level [Figures 3 and 4]. This linear intraspinal D11/12 and D12/L1 level FDG uptake was attributed in our case to possible FDG accumulation and improper clearance from vascular pool of normal course of artery of Adamkiewicz. Accumulation and improper FDG clearance from vascular pool has been cited in literature.[4,5] It has also been reported that ^18^F-FDG accumulation is seen relatively frequently in the arterial tree of cancer patients.[4] Certain medications like potent anti-inflammatory drugs, dexamethasone, prednisone, and tacrolimus[4] can also produce significant vascular ^18^F-FDG uptake. Patient is now clinically asymptomatic and has been advised “Watchful waiting”.

## DISCUSSION

Numerous investigators have reported the incremental value of FDG PETCT in the evaluation of large and medium sized vasculitis.[6] Otherwise gold standard investigation in this condition is histopathology which is not possible in all cases.[7] The main mechanism of action of FDG uptake in vasculitis is thought to be mediated by over expression of GLUT 1 receptor subtype due to macrophage, lymphocytes and neutrophil activation.[8] It produces diffuse FDG uptake in the arterial walls, whereas in atherosclerosis, the uptake is predominantly focal and patchy along the vessels, depending on the stage of the evolution of the atherosclerosis.[8]

Intraspinal FDG uptake was confirmed to be physiological FDG uptake in vessel i.e. artery of Adamkiewicz based on clinical exclusion, CT and MR findings. Normal course of this artery was identified with conventional selective angiography. However, both MR angiography and CT angiography[9,10] have the potential to demonstrate the vessels of interest.

The Adamkiewicz artery is the largest segmental medullary artery typically arising from a left posterior intercostal artery (a branch of aorta) and supplies two thirds of the spinal cord via anterior spinal artery. In 75% of people, the artery of Adamkiewicz originates on the left side of the aorta between the D9 and D11 vertebral segments.[11] In approximately 30% it arises from the right side. 25 % of population can have two large anterior segmental medullary arteries.

## CONCLUSION

With the increasing availability and use of PET scans in our oncological and non oncological practice, it is imperative for us to know all the physiological sites of FDG uptake for confident image interpretation. We can add this intra spinal physiological FDG uptake to the various other listed physiological sites already known.
